# Tuning the structure and properties of a multiferroic metal–organic-framework *via* growing under high magnetic fields†

**DOI:** 10.1039/c8ra00799c

**Published:** 2018-04-12

**Authors:** Lin Hu, Zhe Wang, Hui Wang, Zhe Qu, Qianwang Chen

**Affiliations:** Anhui Province Key Laboratory of Condensed Matter Physics at Extreme Conditions, High Magnetic Field Laboratory, Chinese Academy of Sciences Hefei 230031 Anhui China zhequ@hmfl.ac.cn; Hefei National Laboratory for Physical Science at Microscale, Department of Materials Science & Engineering, Collaborative Center of Suzhou Nano Science and Technology, University of Science and Technology of China Hefei 230026 China cqw@ustc.edu.cn; Key Laboratory of Photovoltaic and Energy Conservation Materials, Institute of Applied Technology, Hefei Institutes of Physical Science, Chinese Academy of Sciences Hefei 230031 China

## Abstract

High magnetic field-induced synthesis has been demonstrated to tune the structure and properties of the multiferroic metal–organic framework [(CH_3_)_2_NH_2_][Mn(HCOO)_3_]. The crystals obtained under 90 kOe exhibit a lower ferroelectric polarization value and reduced magnetic susceptibility, compared to crystals obtained without a field, which is attributed to structural changes induced by the high magnetic field.

## Abstract 2

High magnetic field-induced synthesis has been demonstrated to tune the structure and properties of the multiferroic metal–organic framework [(CH_3_)_2_NH_2_][Mn(HCOO)_3_].

## Graphical abstract

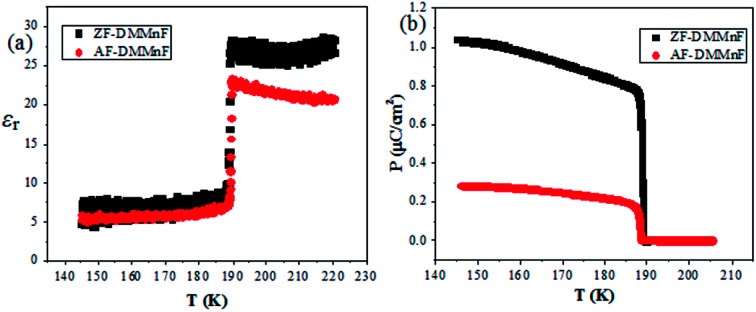

In 2009, Jain *et al.* reported multiferroic behavior in a family of perovskite metal–organic frameworks (MOFs), which stimulated considerable experimental and theoretical efforts to search for new multiferroic materials among perovskite MOFs.^1^ In particular, MOFs with the ABX_3_ perovskite-like structure are of great interest because the variable A and B components provide plenty of room for adjusting the physical and chemical properties in a simple crystalline structure.^2–25^ One such example is formate-based perovskites, [A–M–(HCOO)_3_]. They contain a divalent transition metal ion (M^2+^) which occupies the center of a BO_6_ octahedron, linked by a formate (HCOO^−^) bridging ligand to form a cavity which is occupied by an organic cation. The ordering of hydrogen bonding (N–H⋯O) of the alkylammonium cation triggers the ferroelectic ordering at low temperature, while the weak ferromagnetism arises from metal ions.

It is well known that volatilization and hydrothermal/solvothermal synthesis are the main methods to grow formate-based perovskites, which involve nucleation and growth processes that are affected by physical and chemical factors. Modifying or controlling the physical environment is a simple means to affect the processes of crystallization in order to fine-tune the crystal structure and properties. On the other hand, the effect of pressure on the structure modification of multiferroic MOFs has recently been demonstrated, which showed that ferroelectric polarization could be enhanced by more than three times by applying a compressive strain to the multiferroic frameworks.^14^ Also, density functional theory calculations indicated that changing the magnitude or the canting of the organic molecular dipole can tune the ferroelectric polarization in the new class of multiferroic metal–organic frameworks.^4,15^ These experimental results further highlight the potential of these flexible MOF perovskites to undergo large structural changes in response to an external stimulus.

Crystal growth under a magnetic field is an interesting research topic because a magnetic field may provide a special environment. From the latter half of the 1990s, many studies of magnetic field effects (MFEs) on protein crystallization have been carried out.^26–28^ It has been found that magnetic fields can induce molecular ordering in most organic polymer and biological macromolecules due to the magnetic susceptibility anisotropy of the individual C–C, C–O, C–H, and O–H bonds and their relative orientation in the crystal, or the formation of an interlinked network of biological macromolecules under a high magnetic field.^29–31^ Also, the MFEs on the growth of materials have been extensively studied by our group, which found that both the structure and the properties of materials can be regulated by magnetic fields.^32–38^ For example, it is believed that during Co_3_O_4_ growth, the alignment of the spins and thus the magnetic and crystal lattices of Co_3_O_4_ are influenced by the external magnetic field.^35^ In this paper, we extend the study to grow single crystals of formate-based perovskites [(CH_3_)_2_NH_2_][Mn(HCOO)_3_] (DMMnF) under a high magnetic field. DMMnF is the first prototypical multiferroic formate-based perovskite to be discovered^1^ which exhibits an order-disorder ferroelectric transition below 185 K. It is also a weakly canted antiferromagnet (*T*_c_ = 8.5 K) with a 0.08° canting angle. Moreover, DMMnF is suggested to be thermodynamically more stable than other formate-based perovskites and is easily obtained by a solvothermal process. The main purpose of this study is to show the possibility that the structure and properties of multiferroic MOFs could be tuned by synthesis under high magnetic field, and to try to understand the mechanism. In this work, the growth of DMMnF crystals was carried out with and without an applied magnetic field. The resulting crystals were labeled as AF-DMMnF (applied field) and ZF-DMMnF (zero field), with AF-DMMnF indicating the product obtained under a high magnetic field, and ZF-DMMnF indicating the case without a magnetic field.

The AF-DMMnF and ZF-DMMnF micro-crystal powders (obtained from grinding the as-grown single crystals) and single crystals were characterized by X-ray diffraction (XRD). As shown in Fig. S2a,† the clear sharp peaks of both samples are in good agreement with the theoretical patterns calculated from the single-crystal data.^15^ Moreover, the peaks of the powder samples could be attributed to the diffraction planes of the trigonal phase DMMnF at room temperature, confirming the phase purity of both samples (Fig. S2b†).

Dielectric constant measurements were carried out on single crystals of AF-DMMnF and ZF-DMMnF. The dielectric constants of both samples show a clear anomaly close to 185 K on cooling (Fig. 1a). The shape of the dielectric plot indicates that the samples are undergoing a paraelectric to ferroelectric phase transition, which is consistent with the literature.^1,16^ It should be noted that the dielectric constant of AF-DMMnF is lower than that of ZF-DMMnF above 185 K. Fig. 1b shows the temperature dependence of the electric polarization. Below the phase transition temperature, the electric polarization values of AF-DMMnF are much lower than those of ZF-DMMnF, suggesting that the electric polarization of DMMnF in the ferroelectric state is reduced by growing under a high magnetic field. The highest experimental electric polarizations of AF-DMMnF and ZF-DMMnF within the measured temperature range are ∼0.3 μC cm^−2^ and ∼1 μC cm^−2^, respectively, as shown in Fig. 1b. It is well known that the ferroelectrics of DMMnF arise from the hydrogen bonds between the DMA^+^ cations and the formate framework. The reduction in electric polarization indicates that the hydrogen bond-related interactions may have changed. As is known, the Raman spectrum is sensitive to changes in chemical bonding. The Raman spectra of the samples at different temperatures are presented in Fig. 2, and the main vibration modes assigned to HCOO^−^ and DMA^+^ have been marked. All bands observed below 300 cm^−1^ can be attributed to the lattice modes (Fig. 2a). It is worth noting that the intensities of *ν*_1_(HCOO^−^), *ν*_2_(HCOO^−^) and *ν*_3_(HCOO^−^) are weakened in AF-DMMnF compared to those in ZF-DMMnF. In contrast, the intensity of *ν*_s_(CNC) at 897 cm^−1^ in AF-DMMnF is increased compared to that in ZF-DMMnF. The obvious weakening in the vibrational modes of the formate ions in AF-DMMnF could presumably be influenced by the strength of the N–H⋯O hydrogen bonds between the DMA^+^ cations and the formate framework.^15^ It is suggested that the band at 2788 cm^−1^ can be unambiguously assigned to the stretching modes of the NH_2_ group which directly involve hydrogen-bonds.^12^ Most importantly, at 140 K, the intensity of *ν*(NH_2_) in AF-DMMnF is much lower than that in ZF-DMMnF, as shown in Fig. 2d. This result, combined with the weakened vibration modes of HCOO^−^, indicates the reduced strength of the hydrogen-bonds in AF-DMMnF, which is responsible for the decrease in polarization values. Several claims have been made that magnetic fields change the physicochemical properties of water or prepared laboratory solutions, by influencing nucleation and growth, chemical equilibria, and so on.^39–41^ An enhancement in the hydrogen-bonded strength under a magnetic field of 100 kOe was observed, which was caused by increased electron delocalization in the hydrogen-bonded molecules.^42,43^ Our case can be considered as an example of MFEs on a hydrogen-bonded structure, although the mechanism has as yet not been adequately explained. Fig. S3† is the optical image of the AF-DMMnF and ZF-DMMnF surfaces. The AF-DMMnF exhibits uniform growth steps, which may be attributed to slower nucleation. However, irregular growth layers are observed on the surfaces of the ZF-DMMnF because of rapid nucleation.^15^

**Fig. 1:**
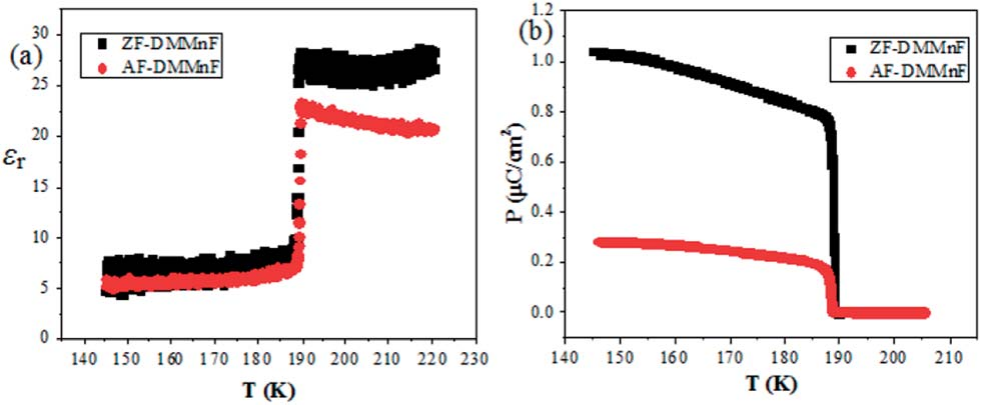
(a) Dielectric constants of AF-DMMnF and ZF-DMMnF as a function of temperature at 1 kHz; (b) the electric polarization of AF-DMMnF and ZF-DMMnF as a function of temperature. Electric polarization was measured after cooling the sample at a voltage of 750V cm^−1^.

**Fig. 2:**
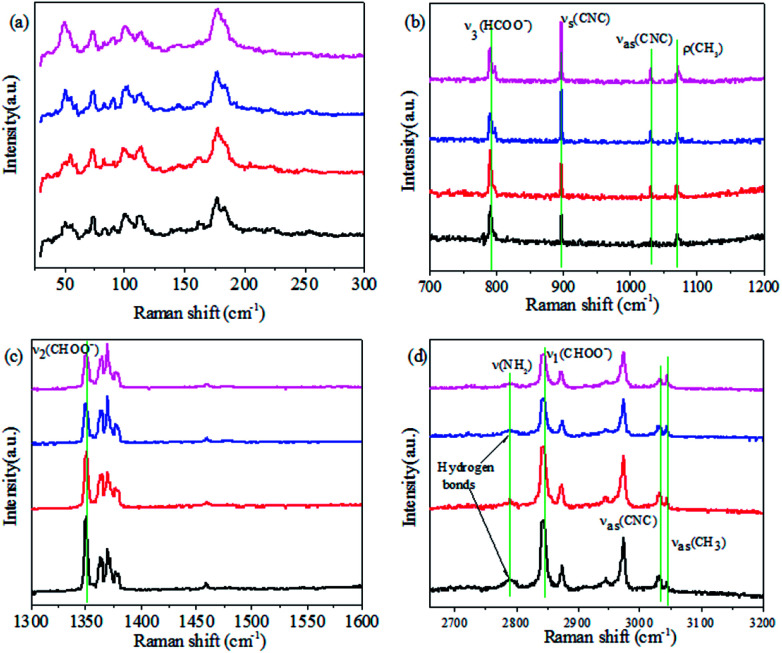
Detail of the Raman spectra corresponding to the spectral ranges 25–300, 700–1200, 1300–1600 and 2650–3200 cm^−1^ at different temperatures for the samples. Black lines: 140 K for ZF-DMMnF; red line: 170 K for ZF-DMMnF; blue line: 140 K for AF-DMMnF; pink line: 170 K for AF-DMMnF.

Wang *et al.* have shown that DMMnF is a canted weak ferromagnet with a *T*_c_ value of 8.5 K.^44^ It is suggested that the spin canting may originate from the noncentrosymmetric character of the three-atom formate bridge, CHOO^−^. Fig. 3a and b show the magnetization as a function of temperature measured at 200 Oe. Both samples show a clear magnetic phase transition at *ca.* 8.5 K. Furthermore, the isothermal magnetization *M*(*T*, *H*) at 1.8 K is shown in Fig. 3c. The magnetization increases almost linearly from 0 to 10 kOe. It is worth mentioning that the magnetic susceptibilities of AF-DMMnF are lower than those of ZF-DMMnF. In the low-field range at 1.8 K, a hysteresis loop can be observed with remnant magnetizations *M*_R_ = 0.0069 μB and 0.0045 μB for ZF-DMMnF and AF-DMMnF, respectively. The canting angle *α* is related to *M*_R_ and *M*_S_ through sin(*α*) = *M*_R_/*M*_S_ (*M*_S_ = 5 μB for a spin-only Mn^II^ ion). In ZF-DMMnF, the canting angle *α* is estimated to be about 0.08°, which is consistent with the reported result.^44^ However, the canting angle *α* in AF-DMMnF is reduced to 0.05° according to the above formula. In a canted antiferromagnetic material, neighboring spins do not align in a strictly parallel manner, but cant each other at a certain angle. During the growth process, the applied magnetic field may disturb this manner and change the angles between the directions of neighboring spins. The decrease in canting angle in AF-DMMnF will generate fewer uncompensated spins, leading to reduced magnetization. This result proves that growth under a magnetic field may be used to control the degree of spin-canting, and then influence the magnetic properties.

**Fig. 3:**
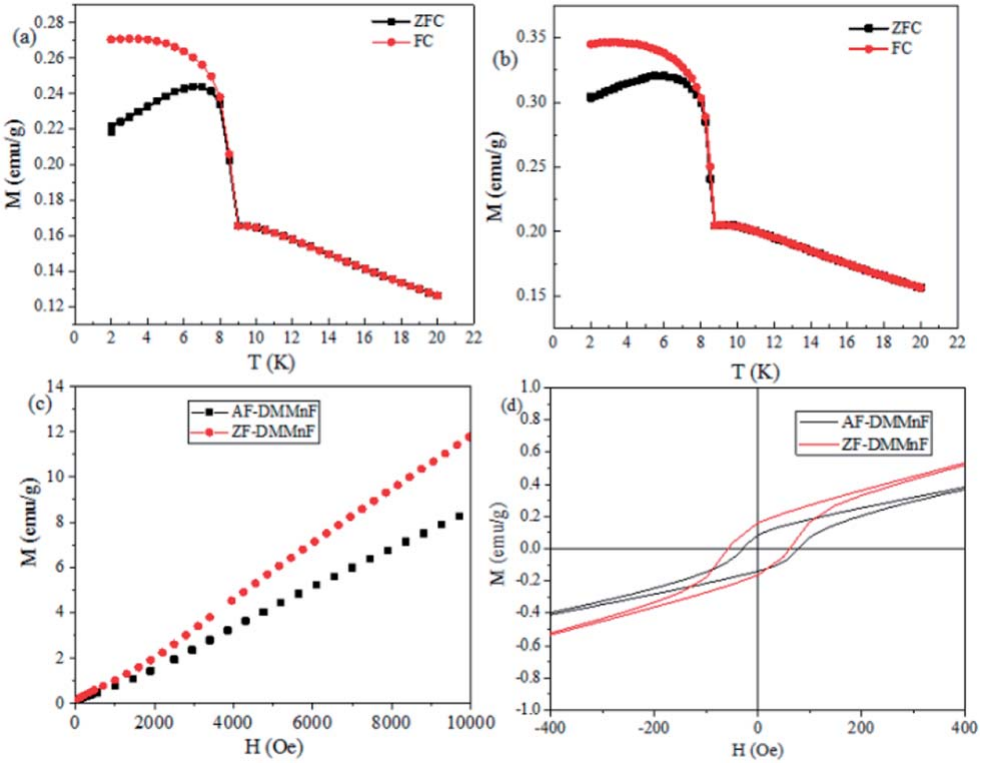
Temperature dependence of *χ*_M_ of AF-DMMnF (a) and ZF-DMMnF (b) measured at 200 Oe from 2 to 20 K (ZFC and FC). (c) Field-dependent isothermal magnetization *M*(*T*, *H*) for AF-DMMnF and ZF-DMMnF at 1.8 K from 0 to 10 kOe. (d) The hysteresis loop measured at 1.8 K.

To further investigate the magnetic states in ZF-DMMnF and AF-DMMnF below *T*_c_, a low temperature electron spin resonance (ESR) technique is employed. ESR embodies the internal environment (crystal field and internal magnetic field) of magnetic ions. Both samples possess a temperature-independent peak and another peak which moves to a low magnetic field when the temperature decreases (Fig. 4). The temperature-independent peak comes from the transition between the Zeeman energy levels of *S* = ±1/2, and the low-field peaks are derived from the transition between the Zeeman energy level of *S* = 1/2 and that of *S* = 3/2 (zero splitting energy, *D* < 0) or *S* = −1/2 and *S* = −3/2 (*D* > 0).^45^ Interestingly, the separation distances between the two peaks of AF-DMMnF are smaller than those of ZF-DMMnF (Fig. 4), indicating the increased zero splitting energies in AF-DMMnF. Moreover, additional weak peaks (circled) appear in the ESR spectrum of ZF-DMMnF, which are probably caused by a small number of magnetic domains in different directions. In other words, the magnetic field makes the magnetic domains of AF-DMMnF more consistent, which will contribute to the dielectric constants and electric polarization values. However, the slight contribution of the domains cannot compensate for the decreases in dielectric constant and electric polarization values induced by the reduction in strength of the hydrogen-bonds. Therefore, AF-DMMnF exhibits a reduced dielectric constant and electric polarization. The increased zero splitting energies, combined with more consistent magnetic domains, further proves that the structure of DMMnF can be regulated by growth under high magnetic fields.

**Fig. 4:**
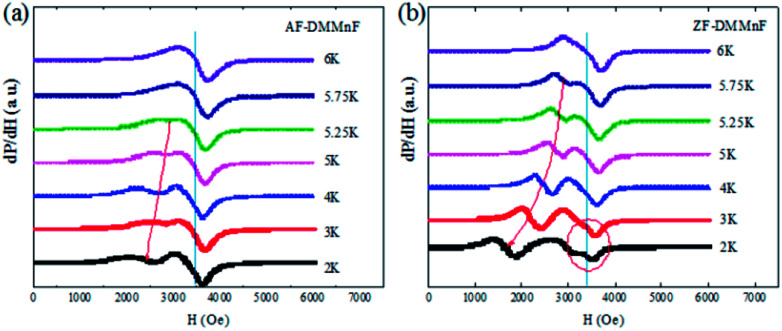
The ESR spectra of AF-DMMnF (a) and ZF-DMMnF (b) at selected temperatures.

In summary, for the first time, it has been demonstrated that the structure and properties of a multiferroic MOF can be tuned by growth under a high magnetic field. The crystals obtained under a high magnetic field exhibit a much lower electric polarization value, which is attributed to the reduced strength of the hydrogen bonds. Moreover, a decrease in magnetic susceptibilities and remnant magnetization was observed due to the reduction in the spin canting angle. With the emergence of superconducting technology, a high magnetic field could be obtained easily, which would open a possible way to tailor the structures and properties of multiferroic MOFs by high magnetic field-induced synthesis.

## Conflicts of interest

There are no conflicts to declare.

## Supplementary Material

RA-008-C8RA00799C-s001
