# Supplementary material for: Tuning the structure and properties of a multiferroic metal–organic-framework via growing under high magnetic fields
Source: RSC Adv. 2018 Apr 12;8(25):13675–8. doi: 10.1039/c8ra00799c (PMC9079801; doi:10.1039/c8ra00799c)
Supplement: RA-008-C8RA00799C-s001 [file RA-008-C8RA00799C-s001.pdf]

Electronic Supplementary Material (ESI) for RSC Advances.
This journal is © The Royal Society of Chemistry 2018

Supporting information

Experimental

Sample synthesis

All the starting reagents used in this work were commercially available and used without further purification.

Crystals obtained without magnetic fields: A mixture of $MnCl_2$•$4H_2O$ (1 mmol), DMF (6 mL), and H_2O (6 mL) was heated in a Teflon-lined autoclave (25 mL) at 140℃ for three days. After slow cooling to room temperature, some black/red precipitation was removed from the solution through filtering. Block crystals were obtained by evaporating the residual solution at room temperature for about three days.

Crystals obtained with a high magnetic field: A mixture of $MnCl_2$•$4H_2O$ (1 mmol), DMF (6 mL), and H_2O (6 mL) was heated in a Teflon-lined autoclave (25 mL) at 140℃ for three days under a high magnetic field of 90 kOe. After slow cooling to room temperature, some black/red precipitation was removed from the solution through filtering. Block crystals were obtained by evaporating the residual solution at room temperature for about three days. A homogenous central magnetic field up to 90 kOe can be produced by a superconducting magnet with a bore diameter of 100 mm. The autoclave was put in the center of the magnetic field. The temperature in the furnace chamber could be adjusted automatically by the controlling temperature system (Fig.S1). The powder samples were obtained by grinding the single crystal in mortar.

Characterization

The crystal structure was confirmed by the Rigaku-TTR3 X-ray diffractometer using high-intensity graphite monochromatized CuKα radiation. Raman scattering measurements were performed using a Horiba Jobin YvonT64000 Micro-Raman instrument with a torus 532 laser (λ=532nm) as an excitation source in a backscattering geometry. A back-illuminated charge coupled device (CCD) cooled by liquid nitrogen was used to detect the scattered light. Electrical measurements were

performed on a homebuilt Multi Measurement System on a Janis 9Tesla magnet. The ploing electric field was applied by using a Keithley 2410 Sourcemeter. The electric polarization (P) was obtained by integrating polarization current with time. The ESR measurements were performed in a Bruker EMXplus 10/12CW spectrometer at X-band frequencies.

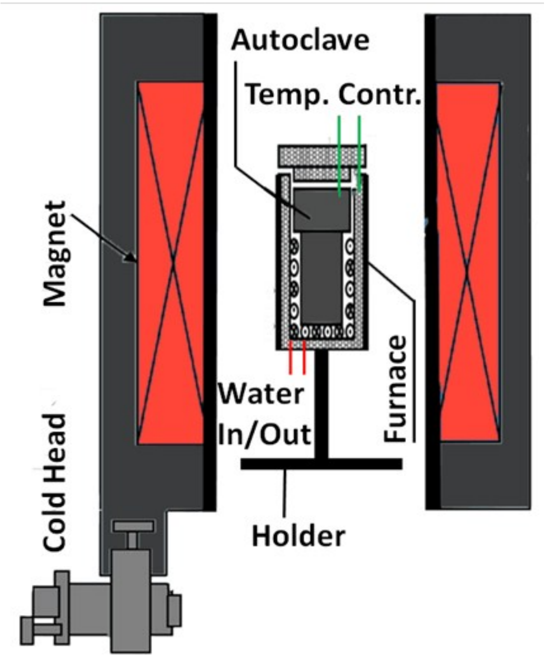

Fig.S1 The schematic illustration of experimental apparatus.

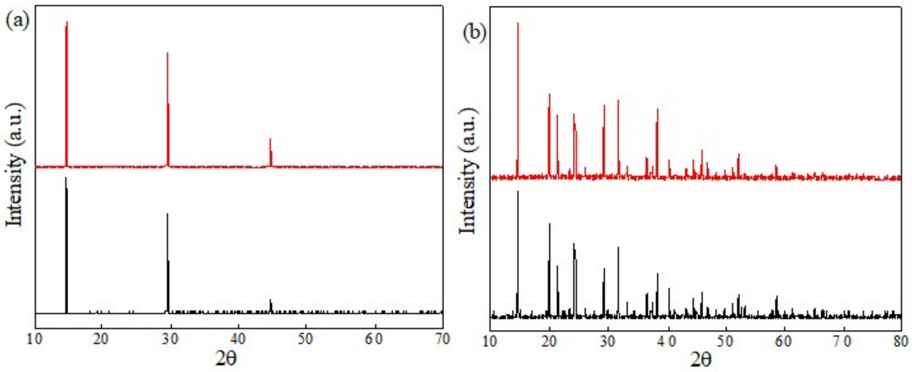

Fig.S2The patterns of the samples (a) single crystal (b) powder. Red line: ZF-DMMnF; Black line: AF-DMMnF.

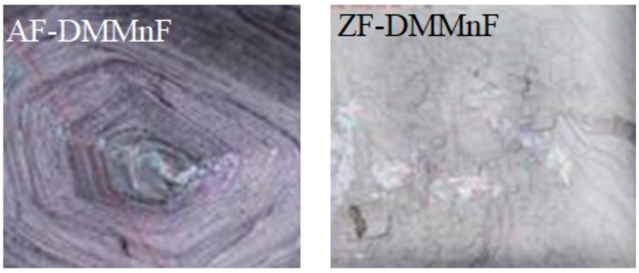

Fig.S3 Optical images of the AF-DMMnF and ZF-DMMnF surfaces.
